# Two New Metabolites from the Endophytic Fungus *Alternaria* sp. A744 Derived from *Morinda officinalis*

**DOI:** 10.3390/molecules22050765

**Published:** 2017-05-08

**Authors:** Ying Wang, Hong-Xin Liu, Yu-Chan Chen, Zhang-Hua Sun, Hao-Hua Li, Sai-Ni Li, Ming-Li Yan, Wei-Min Zhang

**Affiliations:** 1 State Key Laboratory of Applied Microbiology Southern China, Guangdong Provincial Key Laboratory of Microbial Culture Collection and Application, Guangdong Open Laboratory of Applied Microbiology, Guangdong Institute of Microbiology, Guangzhou 510070, China; wangyinghx@163.com (Y.W.); hxinliu1225@163.com (H.-X.L.); yuchan2006@126.com (Y.-C.C.); sysuszh@126.com; (Z.-H.S.); hhli100@126.com (H.-H.L.); lisn@gdim.cn (S.-N.L.); 2 School of Chemistry and Chemical Engineering, Hunan University of Science and Technology, Xiangtan 411100, China; mlyan@hnust.edu.cn

**Keywords:** *Alternaria* sp., endophytic fungus, *Morinda officinalis*, cytotoxicity, α-glucosidase inhibitory

## Abstract

Two new compounds isobenzofuranone A (**1**) and indandione B (**2**), together with eleven known compounds (**3**–**13**) were isolated from liquid cultures of an endophytic fungus *Alternaria* sp., which was obtained from the medicinal plant *Morinda officinalis*. Among them, the indandione (**2**) showed a rarely occurring indanone skeleton in natural products. Their structures were elucidated mainly on the basis of extensive spectroscopic data analysis. All of the compounds were evaluated with cytotoxic and α-glucosidase inhibitory activity assays. Compounds **11** and **12** showed significant inhibitory activities against four tumor cell lines; MCF-7, HepG-2, NCI-H460 and SF-268, with IC_50_ values in the range of 1.91–9.67 μM, and compounds **4**, **5**, **9**, **10**, **12** and **13** showed excellent inhibitory activities against α-glucosidase with IC_50_ values in the range of 12.05–166.13 μM.

## 1. Introduction

**Figure 1:**
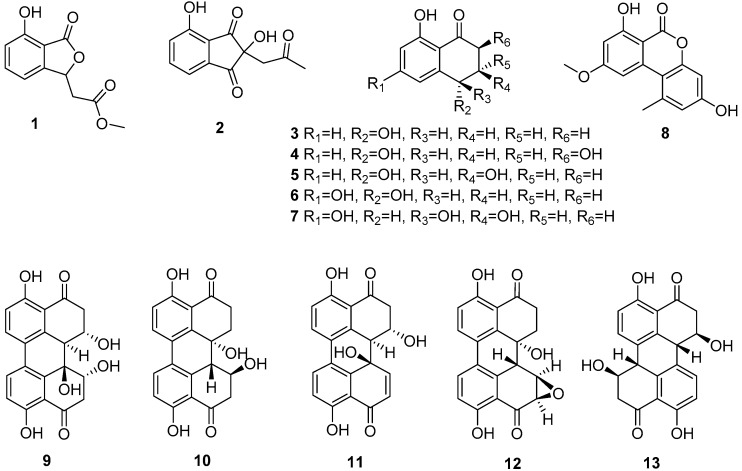
Structures of compounds **1**–**13** isolated from *Alternaria* sp. A744.

Endophytic fungi have been considered a rich source of structurally novel and bioactive diverse metabolites that have become interesting and significant resources for drug discovery [1,2,3]. *Morinda officinalis*, known as one of the ‘top four south authentic traditional Chinese medicines’, has obvious regional characteristics. Its roots contain plant sterols, anthraquinones, flavonoids, vitamin C, sugar, resin and other ingredients, and they are widely used to treat impotence, spermatorrhea, rheumatism and female infertility [4]. However, there are few systematic reports on endophytic fungus resources from this plant and their active components. During our ongoing search aimed at structurally unique and bioactive substances from endophytic fungi [5,6,7,8], we conducted a chemical analysis on a fraction of the broth extract of the fungus *Alternaria* sp. A744 derived from *M. officinalis*, which led to the isolation of two new secondary metabolites along with eleven known compounds (Figure 1). All the compounds were evaluated for their cytotoxic and α-glucosidase inhibitory activities via assays. Herein, the details of the isolation, structural elucidation and bioassay are described.

## 2. Results

### 2.1. Structural Elucidation of New Compounds

**Figure 2:**
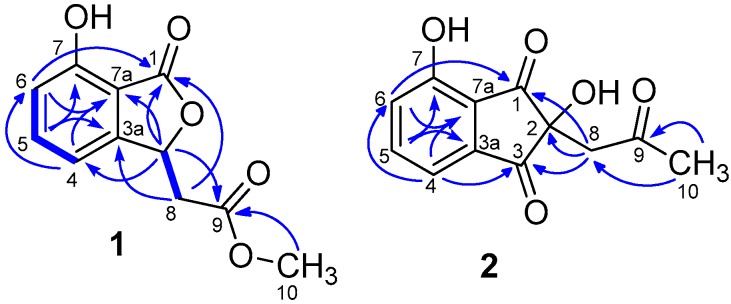
Key ^1^H-^1^H COSY (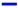) and HMBC (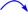) correlations for compounds **1**, **2**.

Isobenzofuranone A (**1**), colorless oil, had a molecular formula of C_11_H_10_O_5_ on the basis of negative high-resolution electrospray ionization mass spectra (HR-ESI-MS) ([M − H]^−^ *m/z* 221.0460, calcd. for 221.0450), corresponding to seven degrees of unsaturation (see Figures S1–S9). The infrared spectroscopy (IR) spectrum exhibited absorption bands at 3435 (hydroxyl group) and 1732 (carbonyl group) cm^−1^. The ^1^H-NMR spectroscopic data of **1** (Table 1) combined with heteronuclear multiple quantum coherence (HMQC) experiment implied one methoxy signal [δ_H_ 3.72 (s, *J* = 1.1, H_3_-10)], one methylene signal [δ_H_ 3.08 (dd, *J* = 16.6, 4.6), 2.80 (dd, *J* = 16.6, 8.0)], one methine [δ_H_ 5.82 (dd, *J* = 8.0, 4.6, H-3)] and three aromatic protons [δ_H_ 6.90 (d, *J* = 8.2, H-6), 7.00 (d, *J* = 7.4, H-4), 7.55 (dd, *J* = 8.2, 7.4, H-5)]. The ^13^C-NMR spectrum revealed 11 carbon resonances attributed to two carbonyl groups (δ_C_ 171.2, 171.5), a methoxyl carbon δ_C_ 52.4 (C-10), one methylene carbon δ_C_ 40.0 (C-8), three sp^2^ quaternary carbons [δ_C_ 158.4 (C-7), 152.2 (C-3a), 112.5 (C-7a)], and three sp^2^ methine carbons [δ_C_ 113.9 (C-4), 137.8 (C-5), 117.1 (C-6)]. The COSY correlations between H-4 (δ_H_ 7.00) and H-5 (δ_H_ 7.55), H-5 and H-6 (δ_H_ 6.9) confirmed the presence of 1,2,3-trisubstituted benzene moiety. The benzene ring along with two carbonyl groups accounted for six unsaturation degrees, while the remaining degrees of unsaturation indicated that an additional ring must be present in the molecule. Meanwhile, a signal of ^13^C-NMR resonance at δ_C_ 171.5 and an absorption in the IR spectrum at 1732 cm^−1^ suggested the presence of a lactone carbonyl group. These data showed a great resemblance to the known compound isoochracinic acid [9], the only difference between them is that a hydroxyl group at position C-10 in the known compound was replaced by a methoxy group in **1**. Furthermore, the heteronuclear multiple bond correlation (HMBC) correlations (Figure 2) from methine proton H-3 (δ_H_ 5.82) to C-1 and C-7a, H_2_-8 to C-1 and C-3a, as well as H-6 to C-7a and C-1 secured the connection of C-3 and C-1 to the aromatic ring. Simultaneously, the relative downfield chemical shift of C-3 (δ_C_ 78.9) revealed a connection with oxygen, thereby forming a lactone ring. The HMBC cross-peaks from H-3 and the proton of methoxy H_3_-10 (δ_H_ 3.72) to C-9 determined the presence of a methyl acetate unit in **1**. The critical COSY signals (Figure 2) from H-3 to H_2_-8 suggested that the methylene group was directly connected to the lactone ring at C-3. Thus, the planar structure of **1** was determined, as shown in Figure 1.

Indandione B (**2**) was obtained as yellow oil. HRESIMS analysis of **2** revealed a molecular formula C_12_H_10_O_5_ ([M − H]^−^ *m/z* 233.0467, calcd. for 233.0450), corresponding to eight degrees of unsaturation (see Figures S10–S17). The IR spectrum exhibited absorption bands at 3377 (hydroxyl group), 1743 and 1703 (carbonyl groups) cm^−1^. The ^1^H-NMR data of **2** (Table 1) revealed one methoxy signal [δ_H_ 2.09 (s, H_3_-10)], one methylene [δ_H_ 3.37 (d, *J* = 2.5, H_2_-8)], and one 1,2,3-trisubstituted benzene moiety [δ_H_ 7.28 (d, *J* = 8.2, H-6), 7.44 (d, *J* = 7.4, H-4), 7.77 (dd, *J* = 7.4, 8.2, H-5)]. The ^13^C-NMR spectrum and the HMQC revealed 12 carbon resonances attributed to one methyl, one methylene, three methines, and seven quaternary carbons. The abovementioned information was quite similar to that of the known compound indanostatin, which was isolated from *Streptomyces* sp. [10]. They all have a typical indandione five-membered ring structure, except for the absence of a hydroxyl group at position C-4 and a methyl group at position C-5 on the benzene ring in **2**. The HMBC correlations from H-4 to C-3, C-6 and C-7a, H-5 to C-3a and C-7, H-6 to C-3a, C-7a, and C-1, along with the HMBC cross-peaks between H-8 to C-1, C-2, and C-3, secured the presence of an indandione five-membered ring. Moreover, the HMBC correlations (Figure 2) from H_2_-8 and H_3_-10 to C-9 implied the presence of a 2-oxopropyl unit in **2**. Finally, the HMBC cross-peaks of methylene protons with C-1, C-2 and C-3 suggested that the 2-oxopropyl group was connected with the indandione five-membered ring at C-2. Therefore, the planar structure of **2** was assigned, as shown in Figure 1.

The known compounds were determined as isosclerone (**3**) [11], 2,4,8-trihydroxy-1-tetralone (**4**) [9], 3,4-dihydro-3,4,8-trihydroxy-1[2*H*]-naphthalenone (**5**) [12], 6-hydroxyisosclerone (**6**) [13], *cis*-4-hydroxyscytalone (**7**) [14], alternariol-4-methyl ether (**8**) [15], 6-*epi*-stemphytriol (**9**) [16], dihydroalterperylenol (**10**) [17], alterperylenol (**11**) [16], altertoxin II (**12**) [18,19], and stemphyperylenol (**13**) [20], by spectroscopic analysis and comparison with previous reports in literature.

### 2.2. In Vitro Cytotoxicity Assay

The in vitro cytotoxic activity of compounds **1**–**13** was investigated against four tumor cell lines, including MCF-7, HepG-2, NCI-H460 and SF-268, by the SRB (Sulforhodamine B) method with cisplatin as the positive control. As outlined in Table 2, compounds **11** and **12** showed significant inhibitory activities against the four tumor cell lines with IC_50_ values in the range of 1.91–9.67 μM.

### 2.3. α-Glucosidase Inhibitory Activity Assay

Simultaneously, all compounds were further evaluated for their α-glucosidase inhibitory activity. Compounds **4**, **5**, **9**, **10**, **12** and **13** showed excellent inhibitory activity against α-glucosidase with IC_50_ values in the range of 12.05–166.13 μM (Table 3), which was obviously stronger than the positive control of acarbose (IC_50_ = 427.34 µM).

## 3. Materials and Methods

### 3.1. General Experimental Material

Optical rotations were determined on an Anton Paar MCP-500 spectropolarimeter (Anton Paar, Graz, Austria) at room temperature. UV spectra were recorded on a Shimadzu UV-2600 spectrophotometer (Shimadzu, Kyoto, Japan). IR spectra were measured using a Shimadzu IR Affinity-1 spectrometer (Shimadzu, Kyoto, Japan). 1D and 2D NMR spectra were performed on a Bruker Avance-500 spectrometer with tetramethylsilane (TMS) as an internal standard (Bruker, Fällanden, Switzerland). ESIMS was measured by an Agilent Technologies 1290-6430A Triple Quad LC/MS (Agilent Technologies, Palo Alto, CA, USA). HRESIMS were done with a Thermo MAT95XP high resolution mass spectrometer (Thermo Fisher Scientific, Bremen, Germany). A Shimadzu LC-20 AT (Shimadzu, Kyoto, Japan) equipped with a SPD-M20A PDA detector was used for HPLC, and a YMC-pack ODS-A/AQ column (250 mm × 20 mm, 5 μm, 12 nm) was used for semi-preparative HPLC separation. Column chromatography (CC): silica gel (200–300 mesh; Qingdao Marine Chemical Inc., Qingdao, China), C-18 reversed phase silica gel (40–63 μm, Merck, Darmstadt, Germany), and Sephadex LH-20 gel (Pharmacia Fine Chemical Co. Ltd., Uppsala, Sweden). Silica gel 60 GF_254_ glass plates (Merck, Darmstadt, Germany) were used for thin layer chromatography (TLC) spotting. All solvents used were of analytical grade (Guangzhou Chemical Regents Company, Ltd., Guangzhou, China). α-Glucosidase from *Saccharomyces cerevisiae* was purchased from Sigma (St. Louis, MO, USA).

### 3.2. Fungal Material

The endophytic fungal strain A744 was isolated from the twigs of *Morinda officinalis*, which was collected from Gaoyao city, Guangdong province of China, in January 2015. The strain A744 was identified by sequence analysis of rDNA ITS (internal transcribed spacer) region. The sequence of the ITS region of the strain has been submitted to GenBank (Accession No. KF706672). By using BLAST (nucleotide sequence comparison program) to search the GenBank database, A744 has 100% similarity to *Alternaria* sp. MY-2011 (Accession No. JN038490). The strain was preserved at the Guangdong Provincial Key Laboratory of Microbial Culture Collection and Application, Guangdong Institute of Microbiology.

### 3.3. Fermentation, Extraction and Compound Isolation

The fungal strain *Alternaria* sp. A744 was cultivated on potato-dextrose agar (PDA) medium at 28 °C for 5 days, and then plugs of agar supporting mycelial growth were cut into small pieces and transferred aseptically into five Erlenmeyer flasks (500 mL), each containing 250 mL potato-dextrose broth, and incubated on a rotary shaker for 5 days at 28 °C at 120 r/min to prepare the seed culture. Then, 10 mL of the seed culture was inoculated into a total of 600 Erlenmeyer flasks (500 mL) containing 250 mL culture broth for 7 days under the same conditions. The culture (150 L) was filtered to separate the broth and mycelia. The broth was extracted five times with EtOAc, while the mycelia were homogenized and saturated with MeOH by the ultrasonic extraction method. The EtOAc crude extract (7.1 g) was subjected to column chromatography (CC) on Sephadex LH-20 (CH_2_Cl_2_/MeOH, 1:1, *v*/*v*) to obtain four fractions (A–D) based on TLC monitoring.

Fraction D (3.5 g) was further purified on C_18_ reversed phase silica gel and eluted with a gradient of MeOH/H_2_O (*v*/*v*, 3:7→10:0) to yield eight major fractions (D1–D8). Subfraction D2 (1.7 g) was separated by a silica gel CC to give five fractions (D2.1–D2.5). D2.2 was subjected to a semi-preparative HPLC (ACN/H_2_O, 30:70, 3 mL/min), then a secondary preparation HPLC (MeOH/H_2_O, 40:60, 3 mL/min) to afford compounds **1** (4.6 mg) and **3** (8.0 mg). D2.3 was further separated by Sephadex LH-20 (CH_2_Cl_2_/MeOH, 1:1, *v*/*v*), following a silica gel CC (Hexane/Acetone, 8:1→2:1, *v*/*v*) and a semi-preparative HPLC (ACN/H_2_O, 15:85, 3 mL/min) to obtain compounds **2** (6.0 mg), **4** (8.4 mg) and **5** (2.2 mg). D2.4 was separated by Sephadex LH-20 (Acetone), following a silica gel CC to yield compound **6** (9.2 mg). D2.5 was separated by a silica gel CC, following a Sephadex LH-20 (Acetone) and a preparation HPLC (ACN/H_2_O, 10:90, 3 mL/min) to afford compound **7** (2.7 mg). D3 (125.3 mg) was further purified on Sephadex LH-20 (MeOH), following a semi-preparative HPLC (MeOH/H_2_O, 55:45, 3 mL/min) to obtain compound **9** (17.0 mg). D4 (101.1 mg) was applied onto a semi-preparative HPLC (MeOH/H_2_O, 45:55, 3 mL/min) to acquire compound **10** (13.4 mg). D5 (46.9 mg) was displayed in the same way as D4 to give compound **11** (20.5 mg). Fraction D7 (26.3 mg) was subjected to a silica gel CC, following a Sephadex LH-20 (Acetone) to obtain compound **12** (2.0 mg). Compound **8** (3.0 mg) was separated from D8 (66.1 mg) by a silica gel CC and a semi-preparative HPLC (MeOH/H_2_O, 80:30, 3 mL/min). Finally, compound **13** (6.0 mg) was isolated from mycelia using a silica gel CC, following a semi-preparative HPLC (MeOH/H_2_O, 50:50, 3 mL/min).

### 3.4. Spectroscopic Data

*Isobenzofuranone A* (**1**): colorless oil; ${\left[\alpha \right]}_{D}^{25}$ +2.9 (*c* 0.1, MeOH); UV (MeOH) *λ*_max_ (log ε) 211 (4.11), 233 (3.56), 299 (3.38) nm; IR *ν*_max_ 3435, 2955, 2920, 2851, 1732, 1607, 1468, 1285, 1161, 1003 cm^−1^; ESIMS negative *m*/*z* 221.0 [M − H]^−^; HRESIMS *m*/*z* 221.0460 [M − H]^−^ (calcd. for C_11_H_9_O_5_, 221.0450); and ^1^H (500 MHz) and ^13^C (125 MHz) NMR data, see Table 1.

*Indandione B* (**2**): yellow oil; ${\left[\alpha \right]}_{D}^{25}$ –4.3 (*c* 0.1, MeOH); UV (MeOH) *λ*_max_ (log ε) 201 (4.03), 235 (3.02), 334 (3.57) nm; IR *ν*_max_ 3377, 2924, 2853, 1744, 1703, 1601, 1464, 1290, 1177, 1020 cm^−1^; ESIMS negative *m/z* 233.0 [M − H]^−^; HRESIMS *m/z* 233.0467 [M − H]^−^ (calcd. for C_12_H_9_O_5_, 233.0450); and ^1^H (500 MHz) and ^13^C (125 MHz) NMR data, see Table 1.

### 3.5. In Vitro Cytotoxicity Assay

The in vitro cytotoxic activities of compounds (**1**–**13**) were assayed against four human tumor cell lines MCF-7, HepG-2, NCI-H460 and SF-268, with cisplatin as a positive control. Assays were performed by the SRB method [21].

### 3.6. α-Glucosidase Inhibitory Activity Assay

An assay of α-glucosidase inhibitory activity was evaluated according the method previously published in Reference [22].

## 4. Conclusions

In this study, thirteen compounds, including two new ones, were isolated from *Alternaria* sp., an endophytic fungus from *Morinda officinalis*. All the structures were established by extensive spectroscopic analysis. The isolates were evaluated their cytotoxicities against four human tumor cell lines and α-glucosidase inhibitory activity assays. Compounds **11** and **12** exhibited significant inhibitory activities with IC_50_ values in the range of 1.91–9.67 μM. Compounds **4**, **5**, **9**, **10**, **12** and **13** showed excellent inhibitory activity against α-glucosidase, which might be useful for further developing α-glucosidase inhibitor.

## Figures and Tables

**Table 1:** ^1^H-NMR (500 MHz) and ^13^C-NMR (125 MHz) data for **1** and **2** in CD_3_OD.

No.	1		2	
	δ_H_ (*J* in Hz)	δ_C_	δ_H_ (*J* in Hz)	δ_C_
1		171.2, C		200.7, C
2				73.8, C
3	5.82 (dd, 8.0, 4.6)	78.4, CH		200.8, C
3a		152.2, C		143.4, C
4	7.00 (d, 7.4)	113.9, CH	7.44 (d, 7.4)	115.5, CH
5	7.55 (dd, 8.2, 7.4)	137.8, CH	7.77 (dd, 8.2, 7.4)	139.1, CH
6	6.90 (d, 8.2)	117.1, CH	7.28 (d, 8.2)	124.4, CH
7		158.4, C		158.3, C
7a		112.5, C		127.4, C
8	3.08 (dd, 16.6, 4.6)	40.0, CH_2_	3.37 (d, 2.5)	49.5, CH_2_
	2.80 (dd, 16.6, 8.0)			
9		171.5, C		208.1, C
10	3.72 (s)	52.4, CH_3_	2.09 (s)	29.3, CH_3_

**Table 2:** Cytotoxic activity of compounds **1**–**13**.

Compounds	IC_50_ (μM)			
	MCF-7	HepG-2	NCI-H460	SF-268
**1**–**8**	≥100	≥100	≥100	≥100
**9**	35.73 ± 1.61	52.38 ± 2.46	43.31 ± 1.75	49.04 ± 1.84
**10**	≥100	≥100	≥100	≥100
**11**	3.73 ± 0.33	5.30 ± 0.95	5.47 ± 0.26	6.57 ± 0.35
**12**	1.91 ± 0.17	5.63 ± 0.10	9.67 ± 0.22	4.25 ± 0.01
**13**	≥100	≥100	≥100	≥100
Cisplatin	3.09 ± 0.27	1.39 ± 0.18	2.43 ± 0.15	2.37 ± 0.35

**Table 3:** α-Glucosidase inhibitory activities of compounds **4**, **5**, **9**, **10**, **12** and **13**.

Compounds	α-glucosidase (IC_50_, μM)
**4**	34.88 ± 1.59
**5**	102.34 ± 2.45
**9**	141.43 ± 7.66
**10**	74.94 ± 2.70
**12**	12.05 ± 2.06
**13**	166.13 ± 2.81
Acarbose	427.34 ± 12.03

## Supplementary Materials

Supplementary material relating to this article can be accessed online.
