# Supplementary material for: Two New Metabolites from the Endophytic Fungus Alternaria sp. A744 Derived from Morinda officinalis
Source: Molecules. 2017 May 8;22(5):765. doi: 10.3390/molecules22050765 (PMC6154570; doi:10.3390/molecules22050765)
Supplement: Supplementary file 1 [file molecules-22-00765-s001.pdf]

Supplemental data

Two New Metabolites from the Endophytic Fungus *Alternaria* sp. A744 Derived from *Morinda officinalis*

Ying Wang [1,2,†], Hong-Xin Liu [1,†], Yu-Chan Chen [1], Zhang-Hua Sun [1], Hao-Hua Li [1], Sai-Ni Li [1], Ming-Li Yan [2] and Wei-Min Zhang [1,*]

[1]State Key Laboratory of Applied Microbiology Southern China, Guangdong Provincial Key Laboratory of Microbial Culture Collection and Application, Guangdong Open Laboratory of Applied Microbiology, Guangdong Institute of Microbiology, Guangzhou 510070, China; wangyinghx@163.com (Y.W.); hxinliu1225@163.com (H.-X.L.); yuchan2006@126.com (Y.-C.C.); sysuszh@126.com (Z.-H.S.);

[2]School of Chemistry and Chemical Engineering, Hunan University of Science and Technology, Xiangtan 411100, China;

*Correspondence: wmzhang@gdim.cn; Tel.: +86-136-0049-9900; Fax: +86-20-8768-8612

[†]These authors contributed equally to this work.

COTENTS

Fig. S1. ^{1}H NMR spectrum of isobenzofuranone (**1**) in CD_3OD.

Fig. S2. ^{13}C NMR spectrum of isobenzofuranone (**1**) in CD_3OD.

Fig. S3. COSY spectrum of isobenzofuranone (**1**) in CD_3OD.

Fig. S4. HMQC spectrum of isobenzofuranone (**1**) in CD_3OD.

Fig. S5. HMBC spectrum of isobenzofuranone (**1**) in CD_3OD.

Fig. S6. ESIMS spectrum of isobenzofuranone (**1**) in CD_3OD.

Fig. S7. HRESIMS spectrum of isobenzofuranone (**1**) in CD_3OD.

Fig. S8. UV spectrum of isobenzofuranone (**1**) in CD_3OD.

Fig. S9. IR spectrum of isobenzofuranone (**1**) in CD_3OD.

Fig. S10. ^{1}H NMR spectrum of indandione (**2**) in CD_3OD.

Fig. S11. ^{13}C NMR spectrum of indandione (**2**) in CD_3OD.

Fig. S12. HMQC spectrum of indandione (**2**) in CD_3OD.

Fig. S13. HMBC spectrum of indandione (**2**) in CD_3OD.

Fig. S14. ESIMS spectrum of indandione (**2**) in CD_3OD.

Fig. S15. HRESIMS spectrum of indandione (**2**) in CD_3OD.

Fig. S16. UV spectrum of indandione (**2**) in CD_3OD.

Fig. S17. IR spectrum of indandione (**2**) in CD_3OD.

Fig. S1. ^{1}H NMR spectrum of isobenzofuranone (**1**) in CD_3OD.

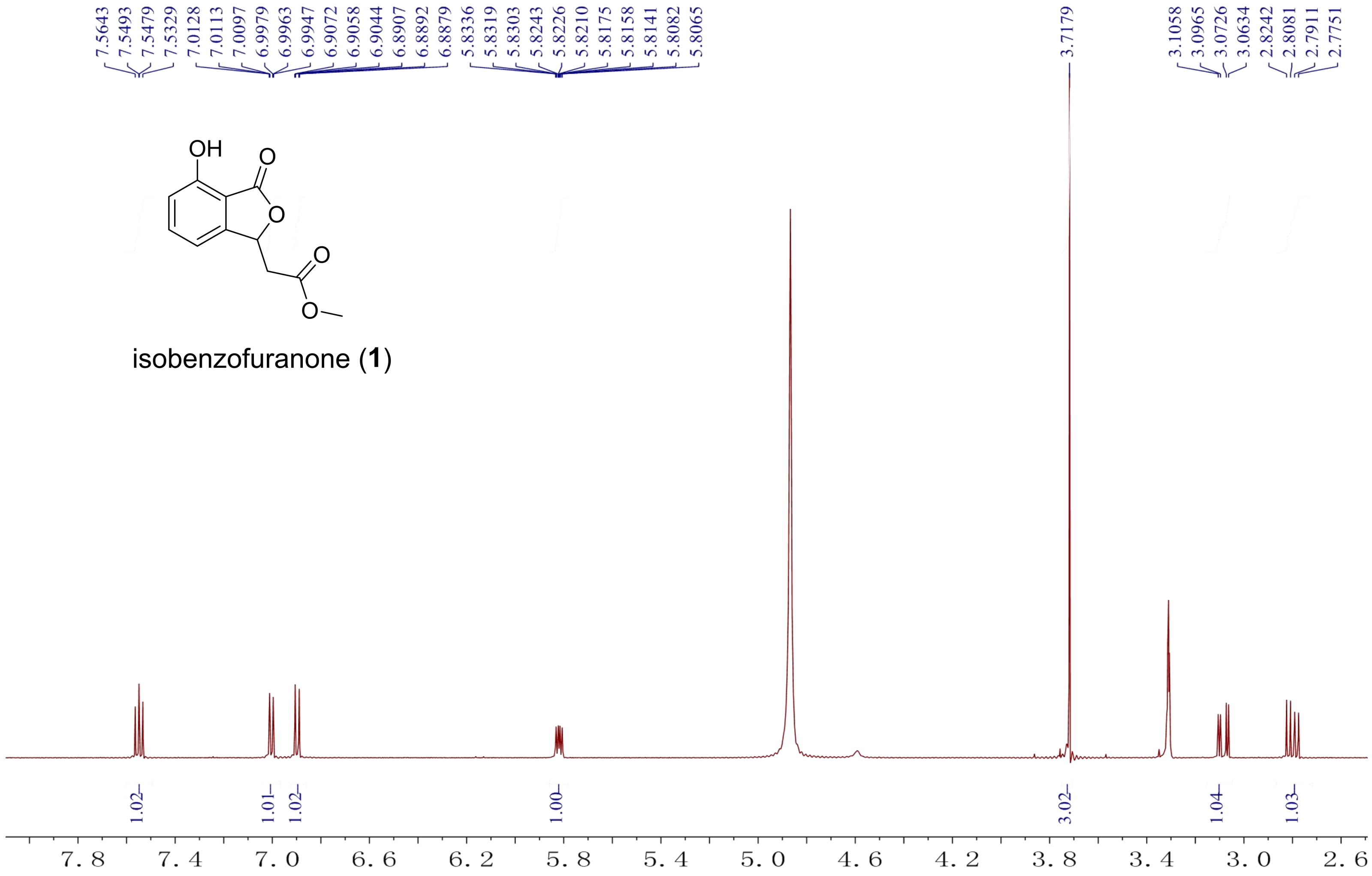

Fig. S2. ^{13}C NMR spectrum of isobenzofuranone (**1**) in CD_3OD.

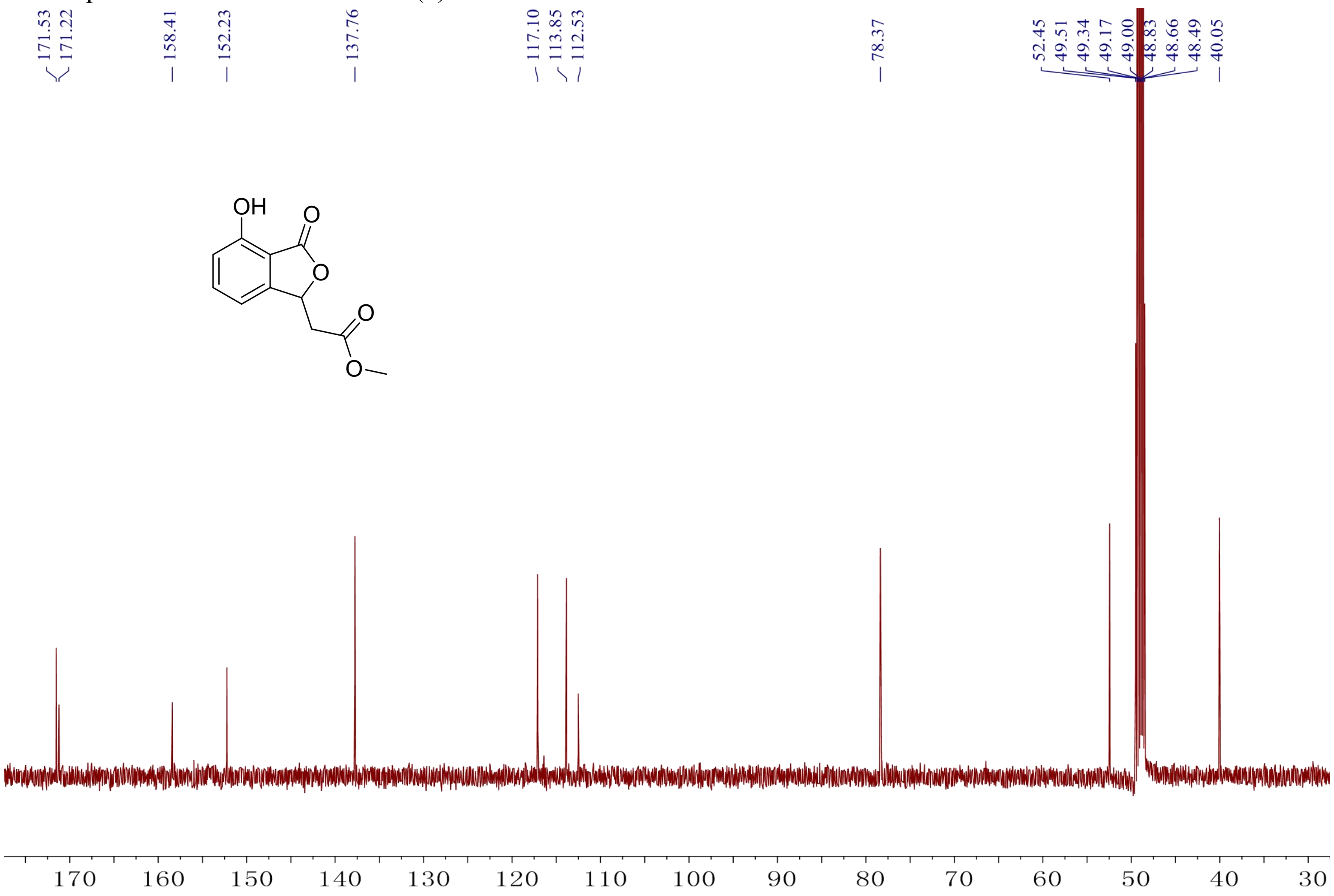

Fig. S3. COSY spectrum of isobenzofuranone (**1**) in CD_3OD.

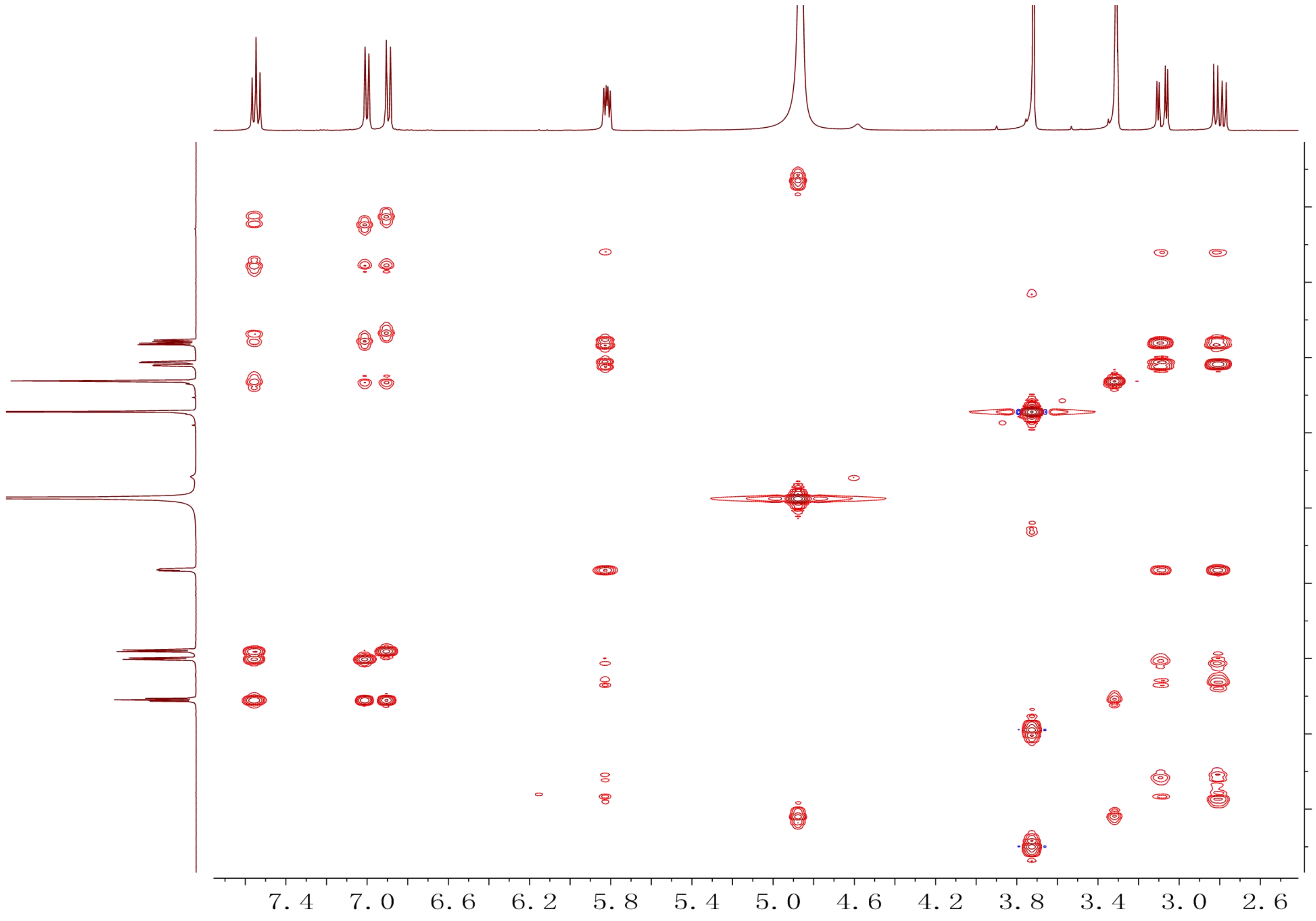

Fig. S4. HMQC spectrum of isobenzofuranone (**1**) in CD_3OD.

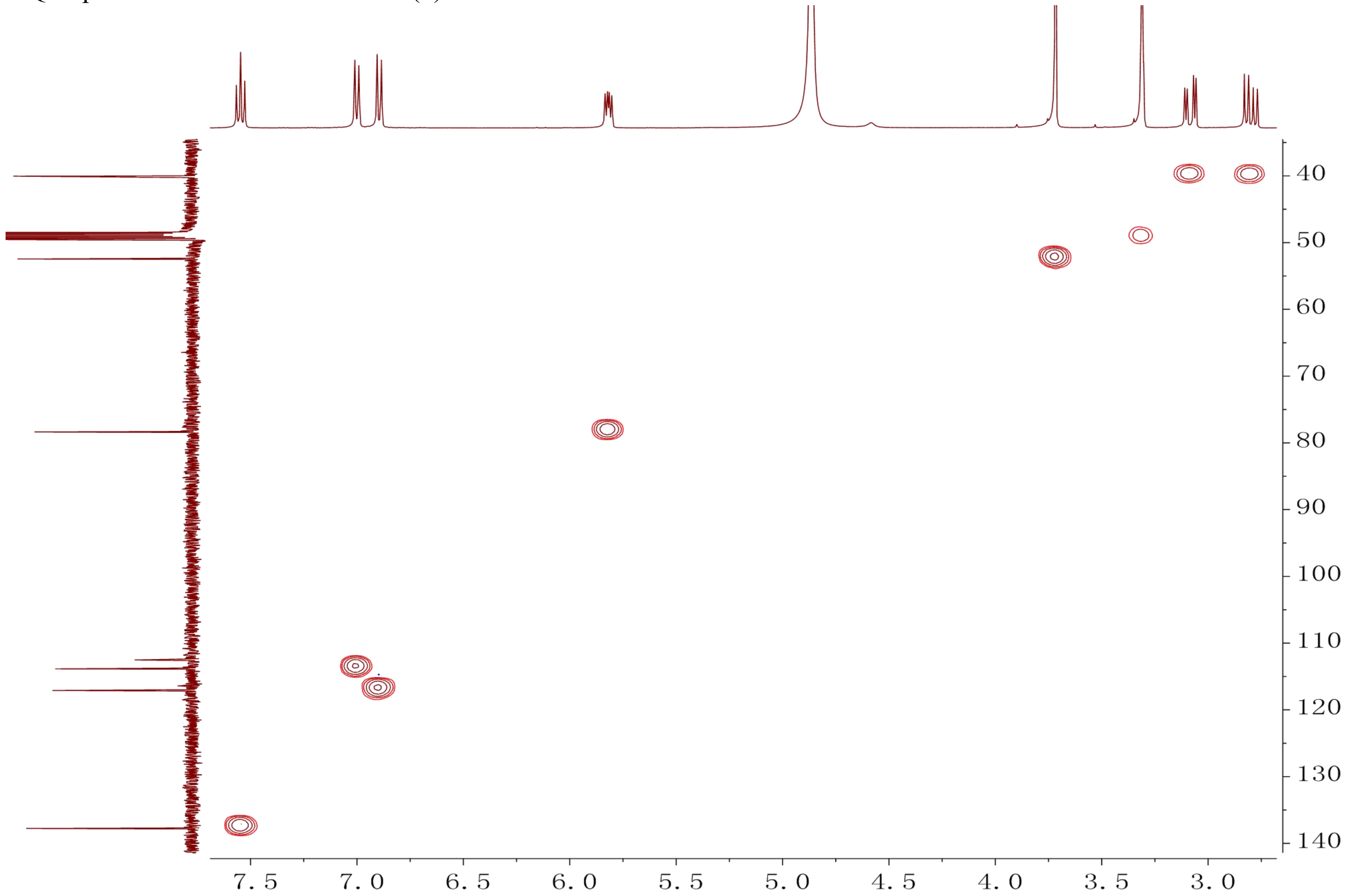

Fig. S5. HMBC spectrum of isobenzofuranone (**1**) in CD_3OD.

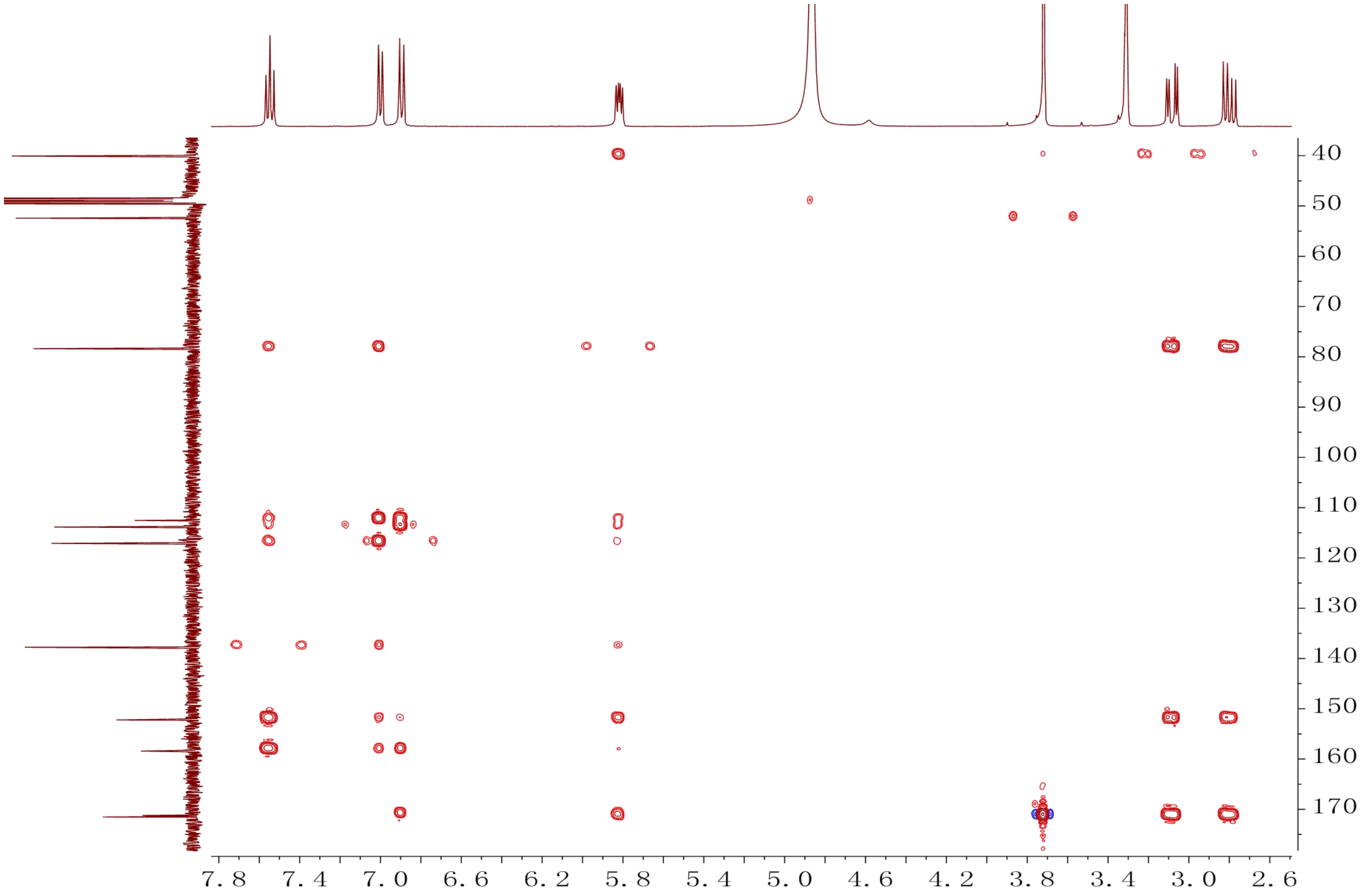

Fig. S6. ESIMS spectrum of isobenzofuranone (**1**) in CD_3OD.

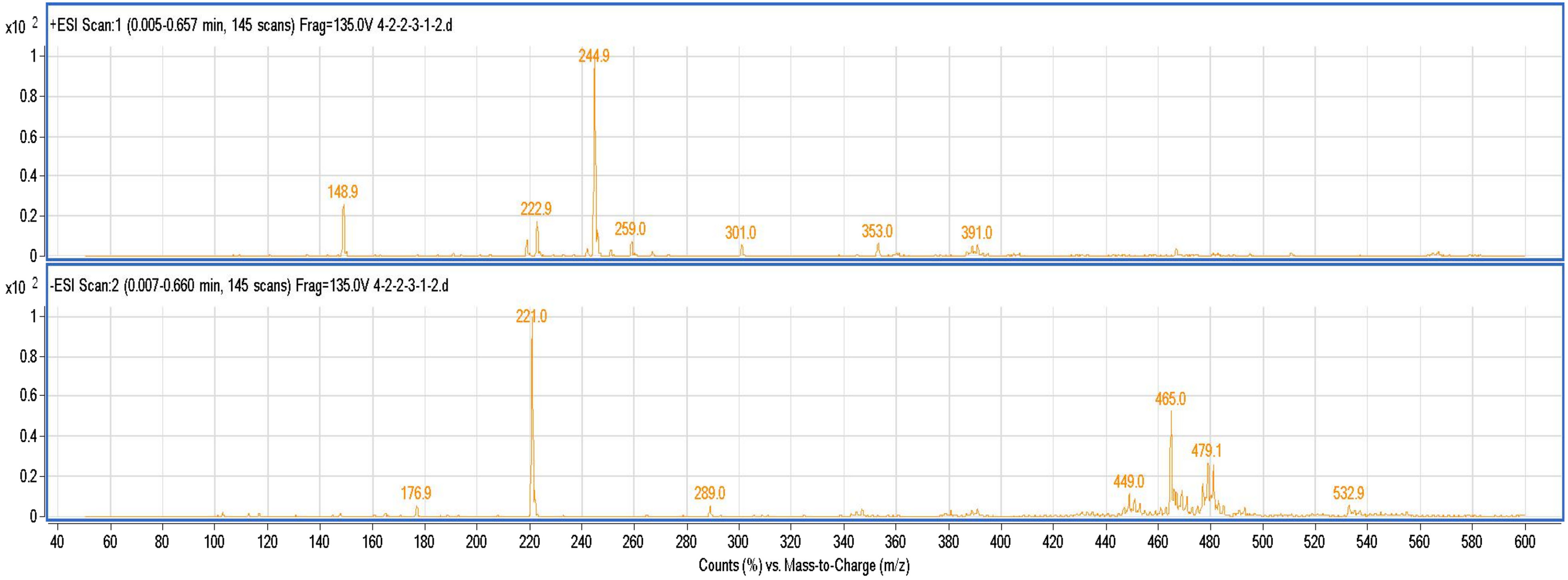

Fig. S7. HRESIMS spectrum of isobenzofuranone (**1**) in CD_3OD.

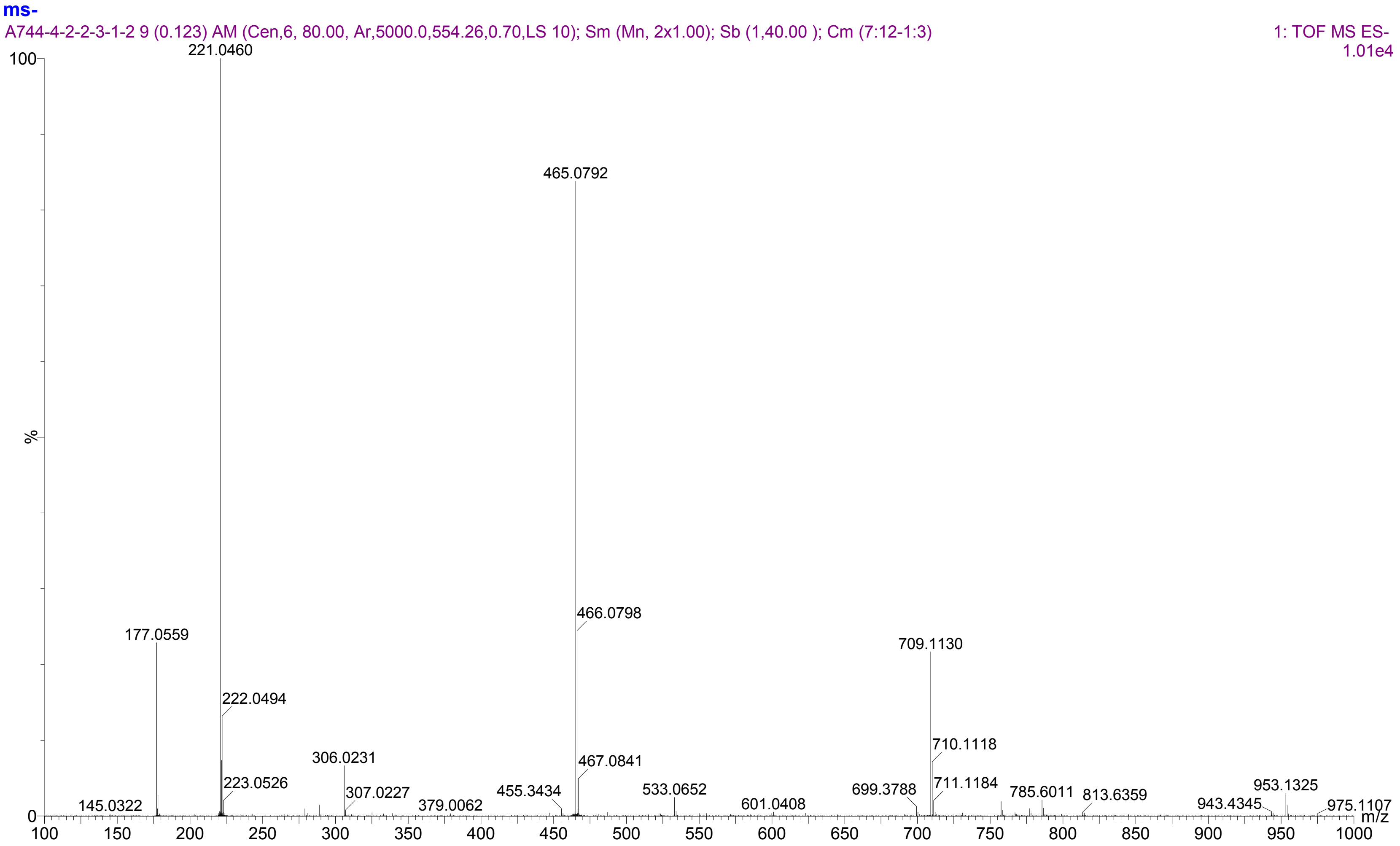

Fig. S8. UV spectrum of isobenzofuranone (**1**) in CD_3OD.

光谱峰值检测报告

2016-10-21 12:12:41

数据集: A744-4-2-2-3-1-2 - RawData

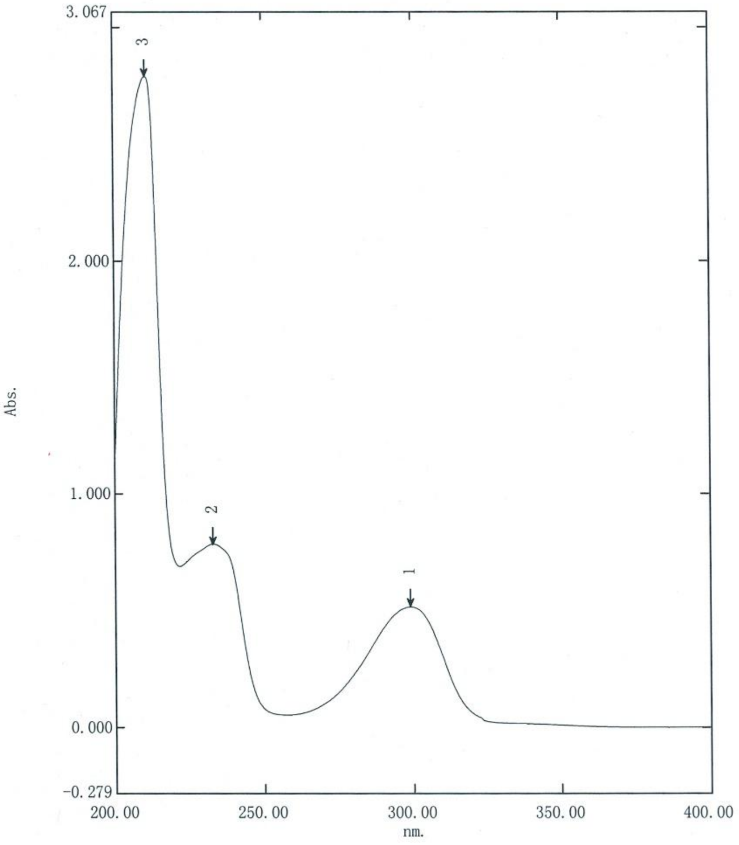

[测定属性]
波长范围 (nm.): 200.00 到 400.00
扫描速度: 中速
采样间隔: 0.2
自动采样间隔: 启用
扫描模式: 单个

[仪器属性]
仪器类型: UV-2600 系列
测定方式: 吸收值
狭缝宽: 2.0
积分时间: 0.1 秒.
光源转换波长: 323.0 nm
检测器单元: 直接
S/R 转换: 标准
阶梯校正: OFF

[附件属性]
附件: 无

[数据处理参数]
阈值: 0.0100000
点: 4
内插: 停用
平均: 停用

[样品准备属性]
重量:
体积:
稀释:
光程长:
附加信息:

No.	P/V	波长(nm)	吸收值	描述
1	↑	299.20	0.516	
2	↑	233.20	0.786	
3	↑	211.00	2.788	

Fig. S9. IR spectrum of isobenzofuranone (**1**) in CD_3OD.

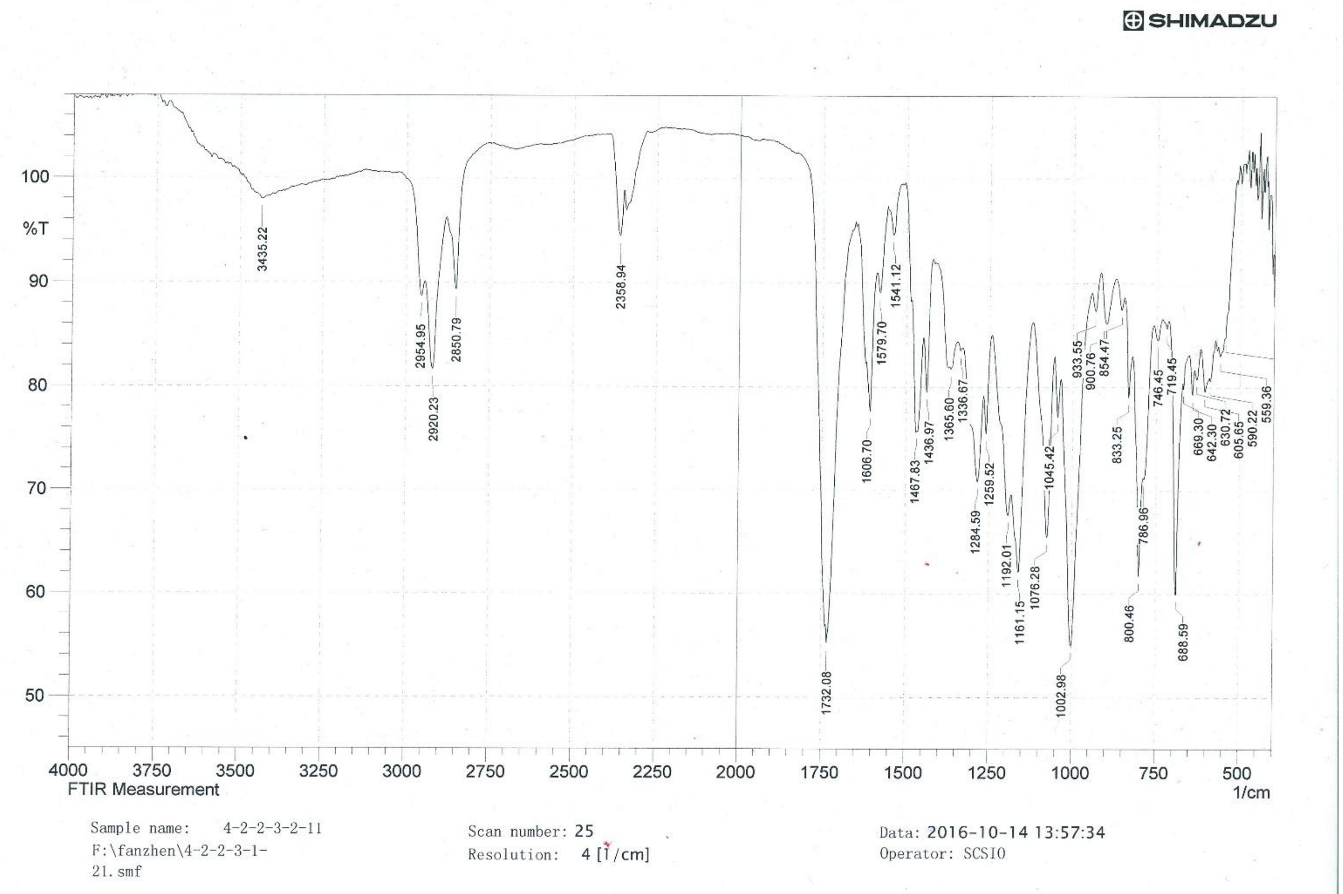

Fig. S10. ^{1}H NMR spectrum of indandione (**2**) in CD_3OD.

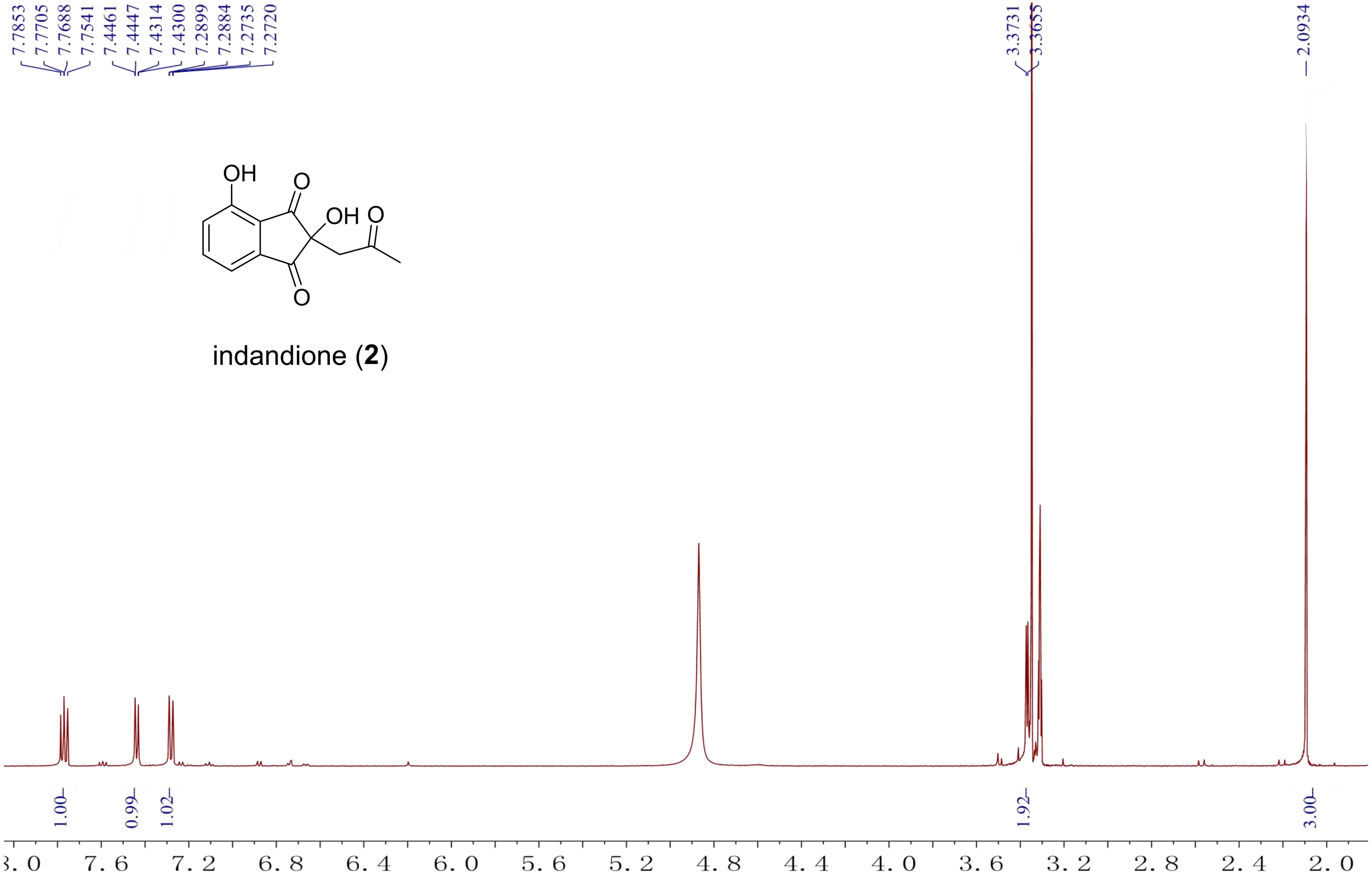

Fig. S11. ^{13}C NMR spectrum of indandione (**2**) in CD_3OD.

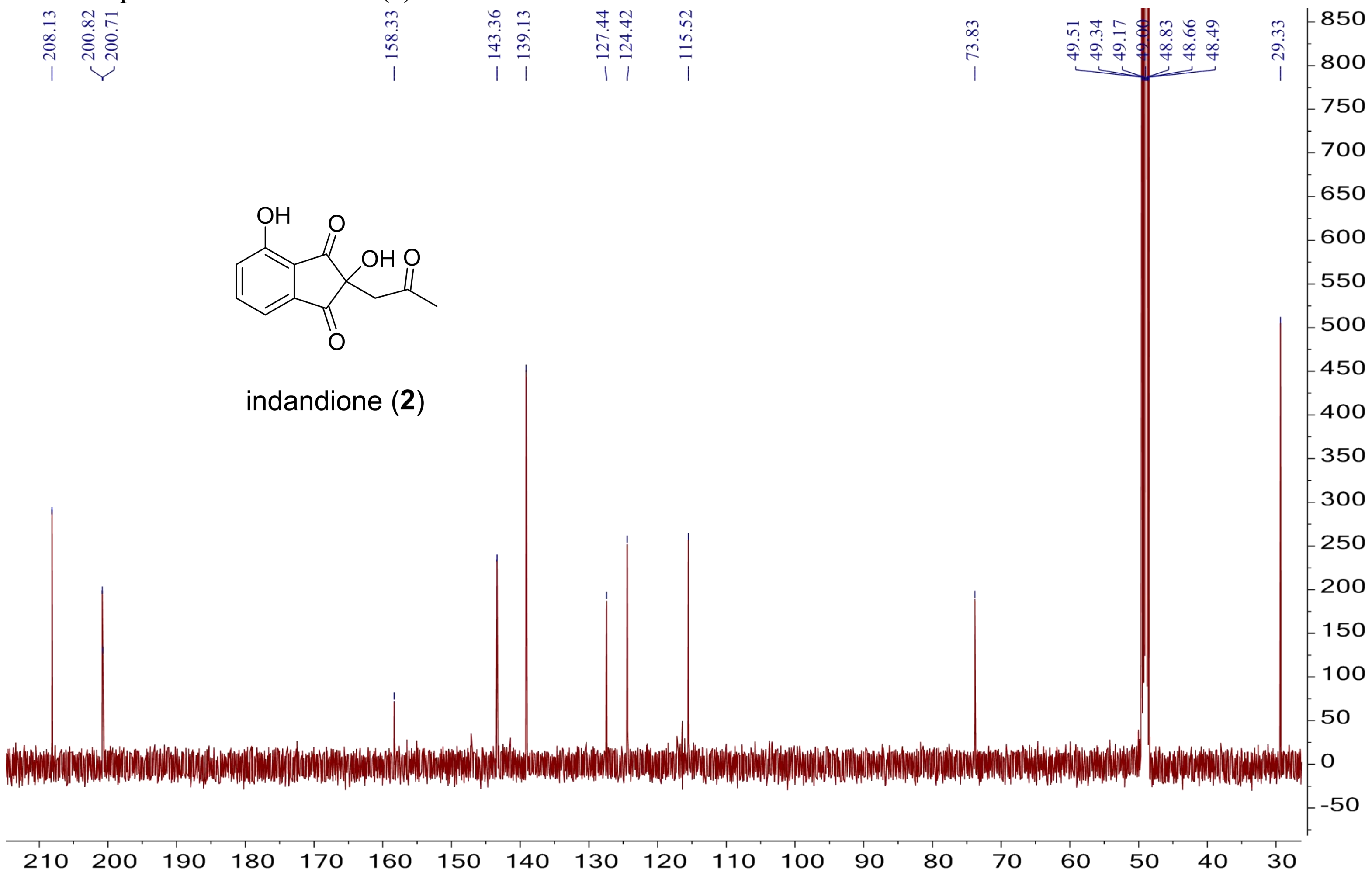

Fig. S12. HMQC spectrum of indandione (**2**) in CD_3OD.

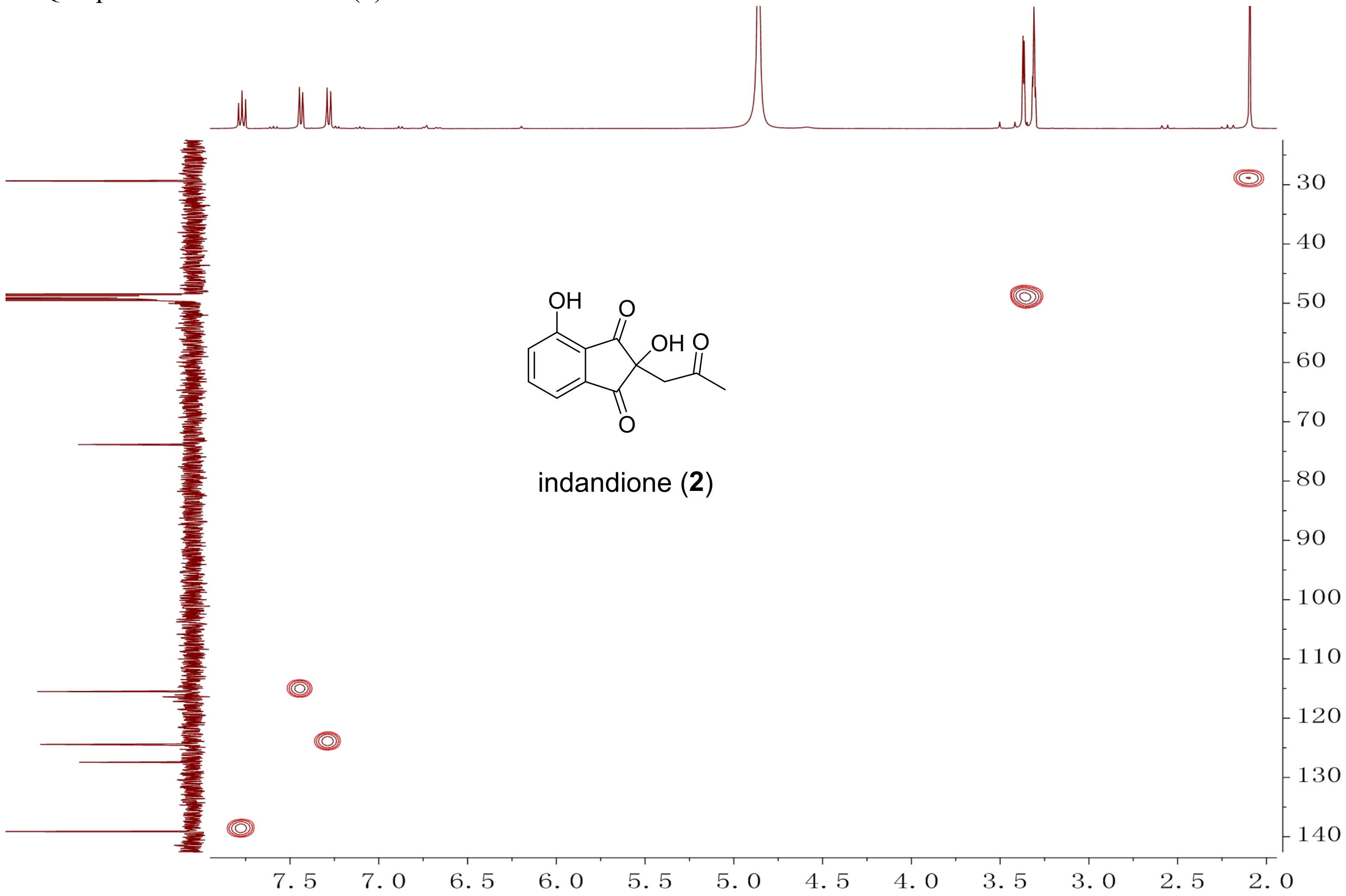

Fig. S13. HMBC spectrum of indandione (**2**) in CD_3OD.

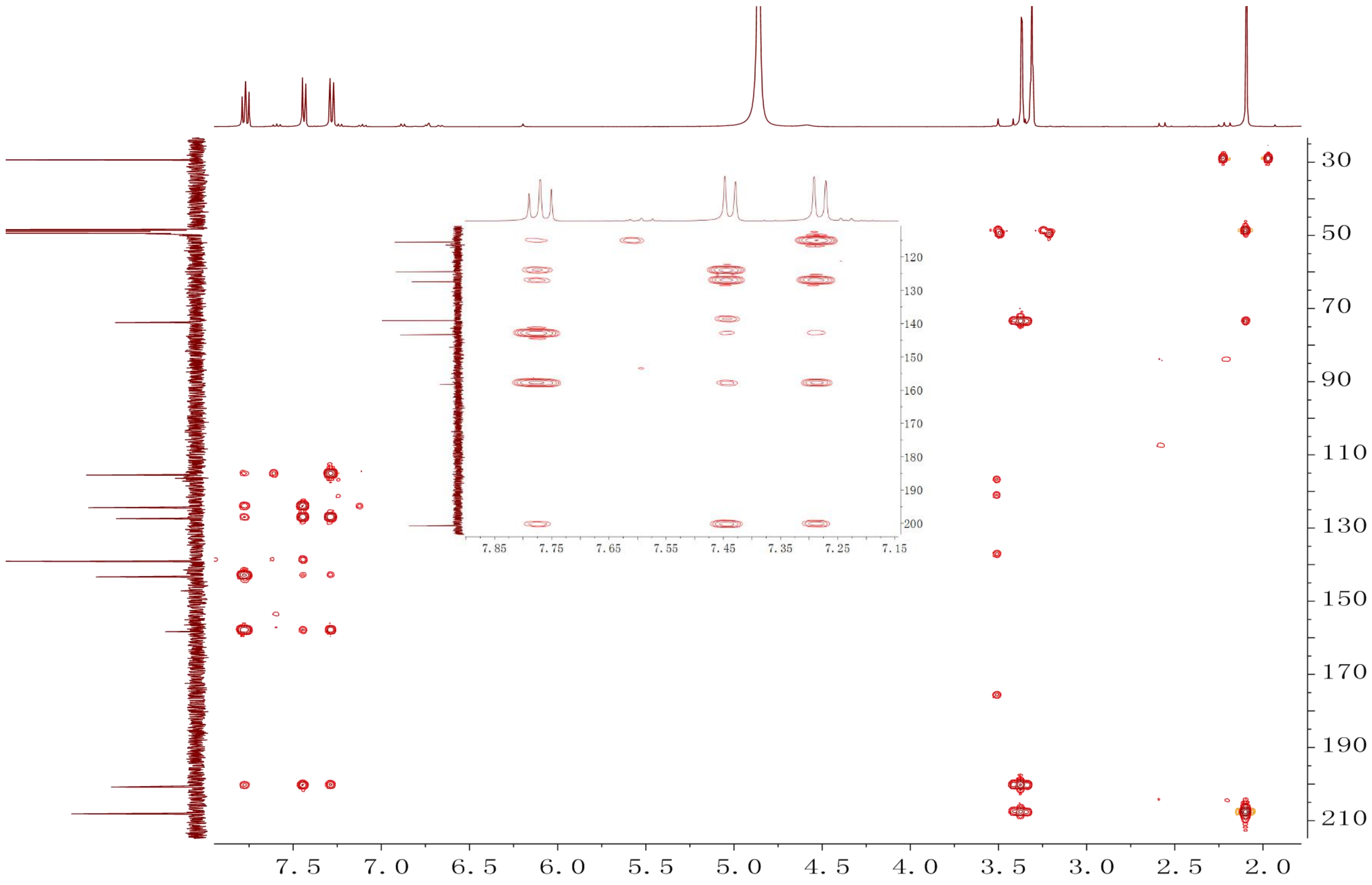

Fig. S14. ESIMS spectrum of indandione (**2**) in CD_3OD.

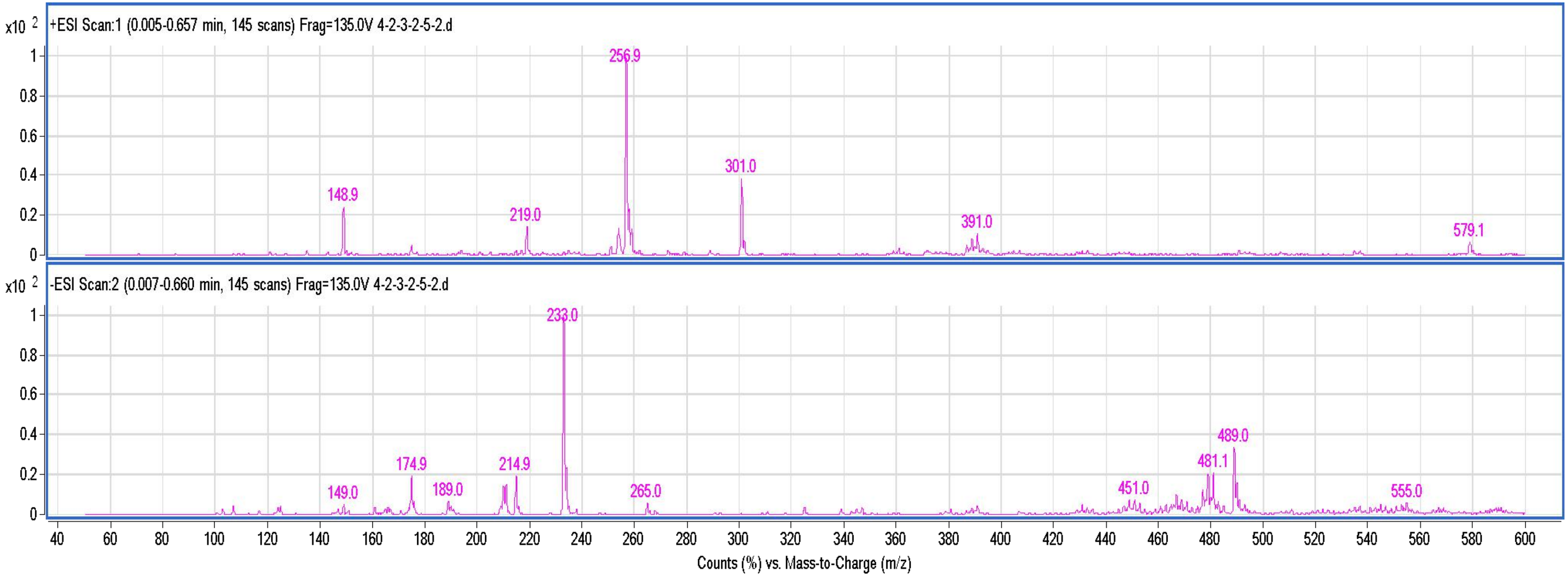

Fig. S15. HRESIMS spectrum of indandione (**2**) in CD_3OD.

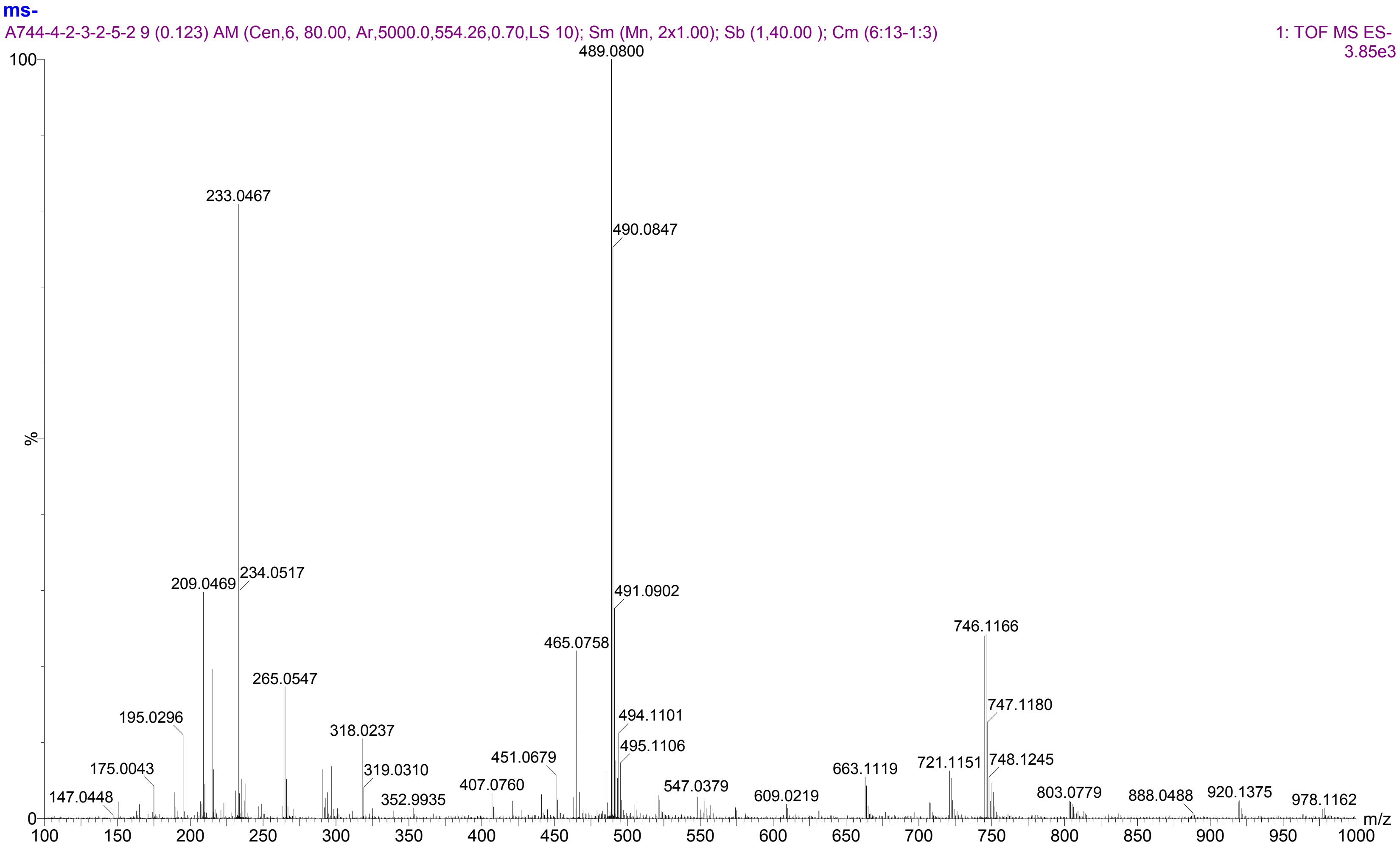

Fig. S16. UV spectrum of indandione (**2**) in CD_3OD.

光谱峰值检测报告

2016-10-21 12:13:03

数据集: A744-4-2-3-2-5-2 01 - RawData

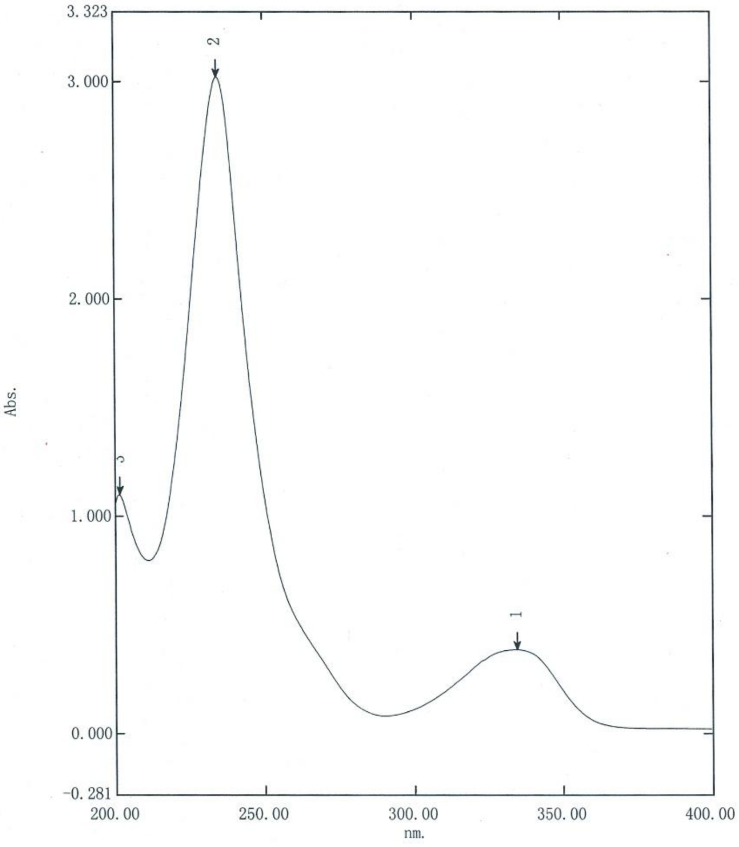

[测定属性]
波长范围 (nm.): 200.00 到 400.00
扫描速度: 中速
采样间隔: 0.2
自动采样间隔: 启用
扫描模式: 单个

[仪器属性]
仪器类型: UV-2600 系列
测定方式: 吸收值
狭缝宽: 2.0
积分时间: 0.1 秒.
光源转换波长: 323.0 nm
检测器单元: 直接
S/R 转换: 标准
阶梯校正: OFF

[附件属性]
附件: 无

[数据处理参数]
阈值: 0.0100000
点: 4
内插: 停用
平均: 停用

[样品准备属性]
重量:
体积:
稀释:
光程长:
附加信息:

No.	P/V	波长(nm)	吸收值	描述
1	↑	334.40	0.384	
2	↑	234.60	3.023	
3	↑	201.40	1.097	

Fig. S17. IR spectrum of indandione (**2**) in CD_3OD.

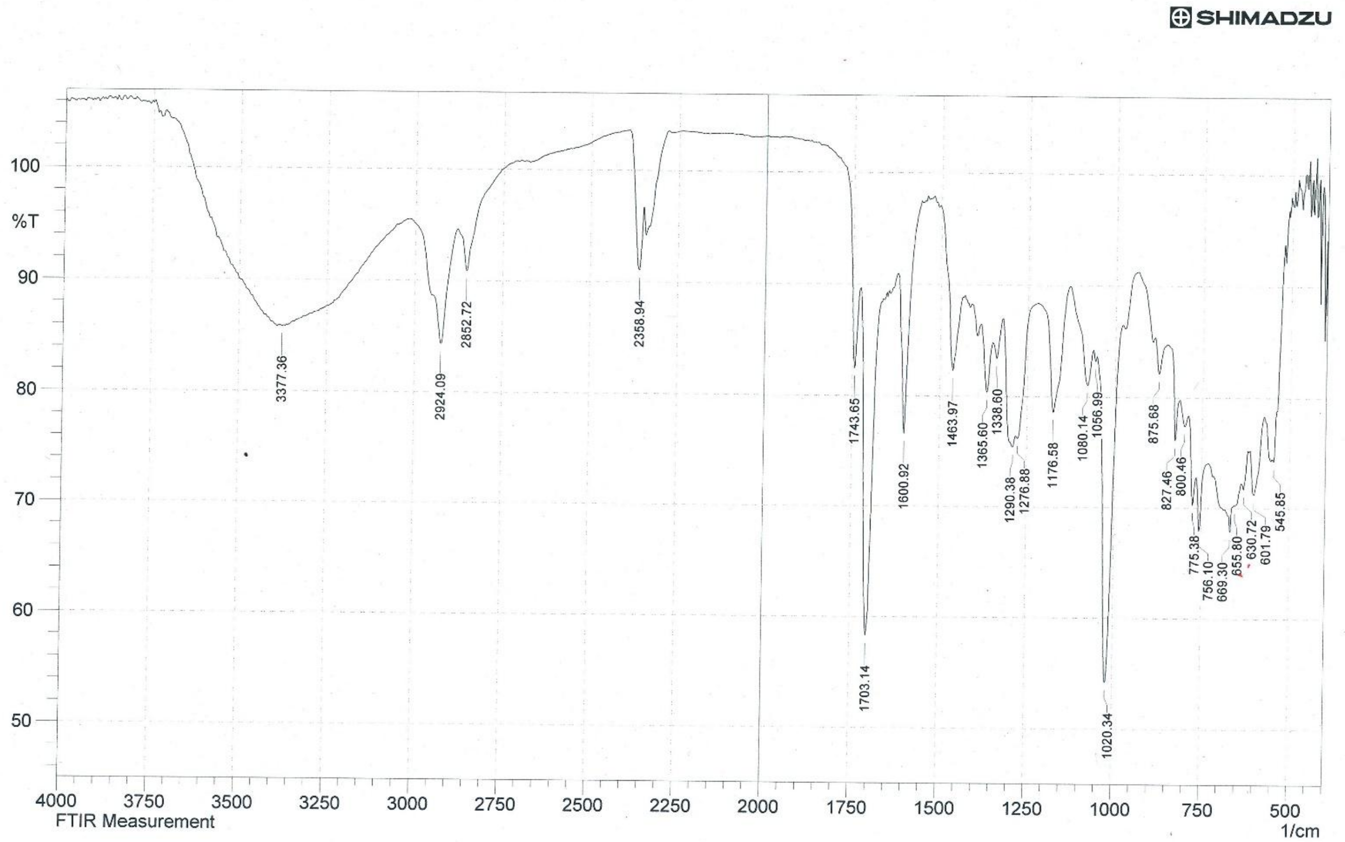

Sample name: 4-2-3-2-5-21
F:\fanzhen\4-2-3-2-5-21.smf

Scan number: 25
Resolution: 4 [1/cm]

Data: 2016-10-14 14:11:30
Operator: SCSIO
